# Impact of the COVID-19 vaccination mandate on the primary care workforce and differences between rural and urban settings to inform future policy decision-making

**DOI:** 10.1371/journal.pone.0287553

**Published:** 2023-06-27

**Authors:** Brigit A. Hatch, Erin Kenzie, NithyaPriya Ramalingam, Eliana Sullivan, Chrystal Barnes, Nancy Elder, Melinda M. Davis

**Affiliations:** 1 Department of Family Medicine, Oregon Health & Science University, Portland, Oregon, United States of America; 2 Oregon Rural Practice-based Research Network, Oregon Health & Science University, Portland, Oregon, United States of America; 3 OHSU-PSU School of Public Health, Oregon Health & Science University, Portland, Oregon, United States of America; University of California Davis School of Medicine, UNITED STATES

## Abstract

**Introduction:**

Little is known about the impact of mandated vaccination policies on the primary care clinic workforce in the United States or differences between rural and urban settings, especially for COVID-19. With the continued pandemic and an anticipated increase in novel disease outbreaks and emerging vaccines, healthcare systems need additional information on how vaccine mandates impact the healthcare workforce to aid in future decision-making.

**Methods:**

We conducted a cross-sectional survey of Oregon primary care clinic staff between October 28, 2021– November 18, 2021, following implementation of a COVID-19 vaccination mandate for healthcare personnel. The survey consisted of 19 questions that assessed the clinic-level impacts of the vaccination mandate. Outcomes included job loss among staff, receipt of an approved vaccination waiver, new vaccination among staff, and the perceived significance of the policy on clinic staffing. We used univariable descriptive statistics to compare outcomes between rural and urban clinics. The survey also included three open-ended questions that were analyzed using a template analysis approach.

**Results:**

Staff from 80 clinics across 28 counties completed surveys, representing 38 rural and 42 urban clinics. Clinics reported job loss (46%), use of vaccination waivers (51%), and newly vaccinated staff (60%). Significantly more rural clinics (compared to urban) utilized medical and/or religious vaccination waivers (71% vs 33%, p = 0.04) and reported significant impact on clinic staffing (45% vs 21%, p = 0.048). There was also a non-significant trend toward more job loss for rural compared to urban clinics (53% vs. 41%, p = 0.547). Qualitative analysis highlighted a decline in clinic morale, small but meaningful detriments to patient care, and mixed opinions of the vaccination mandate.

**Conclusions:**

Oregon’s COVID-19 vaccination mandate increased healthcare personnel vaccination rates, yet amplified staffing challenges with disproportionate impacts in rural areas. Staffing impacts in primary care clinics were greater than reported previously in hospital settings and with other vaccination mandates. Mitigating primary care staffing impacts, particularly in rural areas, will be critical in response to the continued pandemic and novel viruses in the future.

## Introduction

The COVID-19 pandemic increased demand for healthcare services and created a substantial strain on healthcare systems [1]. Thus, maintaining a healthy and robust workforce of healthcare personnel (HCP) was prioritized, particularly given the occupational hazard of infection [1, 2]. Despite safe and effective vaccines against COVID-19 becoming widely available to HCP and other “frontline workers” in early 2021, vaccination rates remained lower than desired [3–5]. To increase rates for HCP, individual and organizational level strategies were implemented [6–10] and many public health policy experts supported COVID-19 vaccination as a condition of employment [11–15]. In the United State (US), six states, including Oregon, adopted HCP vaccine mandates to maintain employment in late 2021; seven additional states mandated vaccination but offered options (e.g., regular testing and masking) for those who refused vaccination and did not discontinue their employment [16]. Oregon’s mandate required vaccination for all HCP (including licensed clinicians, support staff, and any person working in a health care setting). Employ could apply for a religious our medical exemption from this mandate and be considered on a case-by-case basis by their employer. Individuals out of compliance with the mandate were placed on leave or had employment terminated [16, 17]. The US Centers for Medicare and Medicaid required vaccination or exemption by early 2022 for all certified providers, including ambulatory care staff [18, 19].

Mandated vaccination as a condition of employment has strong legal and ethical foundations [17]. HCP have been mandated to receive vaccinations or prove immunity for other infectious diseases, including hepatitis B, influenza, and measles, mumps, and rubella [15, 20]. Influenza vaccination mandates contributed to increased vaccination rates among HCP [7, 8, 21, 22], lower rates of HCP infection [21], and can potentially lead to lower rates of pneumonia and influenza mortality [23]. Small proportions of job loss (0–3%) were reported after influenza vaccination mandates, but studies were limited to hospital settings. The impacts of non-COVID-19 vaccination mandates on outpatient personnel, particularly in rural areas, are not well understood [7, 24].

With regard to COVID-19, vaccination mandates led to minimal job loss in hospitals, rehabilitation centers, and home health agencies reported [25]. In Oregon, major hospital systems reported that vaccination-related job loss impacted <1%-3.5% of employees in the first week after implementation of a statewide mandate [26]. Little has been reported about the impact of these mandates on the primary care workforce, despite primary care providing the majority of healthcare and serving as the frontline for prevention, testing, and treatment of COVID-19 [27]. As COVID-19 vaccine mandates for HCP remain controversial [11, 15, 28–30], understanding their impacts on the primary care workforce is important for future policy decision-making. Rural clinics are of particular interest as they may be prone to amplified workforce challenges due to geographic isolation, difficulties with recruitment, and smaller numbers of total staff [31].

To understand the impact of the COVID-19 vaccination mandate for HCP on the primary care workforce and the differential impact for rural compared to urban clinics, we surveyed clinicians and staff from primary care clinics across one Western state. Learnings will: 1) elucidate the impact of the COVID-19 vaccination mandate in the primary care setting; 2) inform COVID-19 policy decisions as new pandemic variants of concern and additional boosters or vaccinations roll out; and 3) provide important information for future novel viruses or pandemics. Findings are vital given the critical role primary care plays in supporting vaccine and virus response.

## Methods

### Study design and setting

We conducted a cross-sectional mixed-methods survey of clinicians and staff at primary care clinics across Oregon. The study protocol and survey instrument were approved by the IRB at Oregon Health & Science University (IRB #23649).

In Oregon, vaccination against COVID-19 became required (as a condition of employment) for anyone working in a healthcare setting, starting October 18, 2021. Employers were responsible for enforcement and required to document vaccination or waiver (religious or medical) with a civil penalty of $500 per violation per day for employers that failed to comply [32].

### Study participants

A convenience sample of participants were recruited from the listserv of the Oregon Rural Practice-based Research Network (ORPRN). ORPRN is an established practice-based research network of 296 clinics (nearly half of Oregon primary care clinics) [33] that conducts research, education and quality improvement projects with clinics and communities across the state in both rural and urban areas [34, 35]. An email to the ORPRN listserv invited primary care clinicians and staff to participate in a brief electronic survey regarding the impacts of the recent vaccination mandate on their clinic. The listserv includes at least one representative from all 296 clinic partners of ORPRN and for some clinics includes multiple representatives that have participated in prior projects or connected with the network in other ways. The listserv also includes some ORPRN partners working outside of primary care clinics, however, only primary care clinic staff were invited to participate in the invitation contained an information sheet describing the study and explaining that participation was optional. Completion of the survey implied consent. Listserv recruitment was utilized to maximize representation from clinics across the state. Individuals received a reminder email two weeks later. Responses were collected for three weeks total.

### Survey instrument

A 19-item survey requested information about clinic size, location, and staff composition, and multiple choice and open-ended questions about staffing impacts of the vaccination mandate. The survey also included three open-ended questions. The survey was hosted on REDCap (an online platform) [36], accessed from an emailed link, and took 5–10 minutes to complete (see S1 Appendix for complete survey instrument).

### Participants

Clinics were the primary unit of analysis. Surveys were excluded if responses did not contain information for at least two of the four primary outcomes (listed below), if the clinic name was absent, or if the organization did not provide primary care (e.g., skilled nursing facility, specialty care, behavioral health). Most clinics had only one survey respondent, but for 13 clinics with multiple respondents, a single survey was selected to represent the clinic for quantitative analyses. The selected survey was the most complete; or if there were multiple complete surveys per clinic, the selected survey came from the clinic manager, medical director, or a physician/advanced practice provider (APP). The qualitative analysis was conducted at the level of the individual and clustered responses within each clinic.

### Variables

The primary outcomes of interest were the impact of Oregon’s COVID-19 vaccination mandate on clinic-level job loss (*i*.*e*., whether staff were reassigned, put on leave, or had employment terminated), use vaccination waivers, new vaccination of staff, and the perceived significance of the mandate on clinic staffing (see S1 Appendix for specific questions). To account for clinic size in the interpretation of number of staff impacted by job loss, waivers, and newly vaccinated, we created ratios which divided these outcomes by the reported number of clinicians in the clinic. For ease and accuracy of reporting by individual respondents, only the number of clinicians (not total staff) was requested in the survey, and this served as a proxy for overall clinic size.

Outcomes were stratified by clinic-level rurality. Rural clinics had Rural-Urban Commuting Area (RUCA) codes >4 based on the US Department of Agriculture (USDA) guidance or were designated by the Oregon Office of Rural Health using methodology previously employed by our network [37]. Briefly, RUCA codes 1–3 are Metropolitan, 4–5 are Micropolitan, 7–9 are small towns, and 10 are rural areas. The Oregon Office of Rural Health defines any county not part of a Metropolitan area as rural [38, 39].

Categorical outcomes in rural and urban clinics were compared using a Freeman-Halton extension of Fisher’s Exact test, given fewer than 100 clinics in our sample. Comparisons were assessed with two-sample t-tests (continuous) and Mann-Whitney U Test (ordinal). Statistical significance is reported at p<0.05.

### Qualitative analysis

Short answer responses to open-ended questions asking participants to describe the effect of the COVID-19 vaccination mandate on their clinic staff and clinical care, and other thoughts they would like to share were entered into ATLAS.ti Windows (Version 9) [40] and analyzed using a template analysis approach [41]. Five qualitative analysts (MD, EK, NE, NR, CB) read all responses, took individual analytical memos, and collectively created a coding template based on key themes. Then three analysts (NE, NR, CB) coded all responses with an a priori codebook based on template themes. Code-specific review of study data was used to narrow and refine themes. In addition, a matrix was used to compare responses across role type, rurality, and clinic to explore variation in themes based on those variables. Themes were then discussed with the broader analytic team until consensus was reached and final themes were determined.

## Results

143 individuals from 108 clinics returned surveys. Twenty-two surveys (from 13 clinics) were excluded due to incomplete outcome data and 20 surveys (from 15 clinics) were excluded because they did not relate to a primary care clinic, leaving a total of 101 complete surveys from 80 unique clinics (27% clinic-level response rate). Respondents were distributed across geographic regions of Oregon and represented 28 of 36 counties.

### Quantitative results

Table 1 summarizes characteristics of represented clinics which included 34% characterized as small (1–5 clinicians), 29% medium (6–10 clinicians), and 38% large (>10 clinicians). 79% of rural clinics and 48% of urban clinics had small and medium-sized clinics. Most responses came from physicians or APPs: 58% of respondents from rural clinics and 74% from urban clinics were physicians or APPs. 80% of clinics were hiring prior to the vaccination mandate: 60% of rural clinics and 48% of urban clinics were specifically hiring non-clinician staff.

**Table 1:** Characteristics of participating clinics, stratified by rurality.

	Rural n = 38 clinics # (column %)	Urban n = 42 clinics # (column %)	Total n = 80 # (column %)
Clinic size			
Small (1–5 clinicians)*	15 (39.5)	12 (28.6)	27 (33.8)
Medium (6–10 clinicians)*	15 (39.5)	8 (19.0)	23 (28.7)
Large (>10 clinicians)*	8 (21.0)	22 (52.4)	30 (37.5)
Staffing needs prior to mandate			
Hiring clinicians (MD/DO/NP/PA)	10 (26.3)	11 (26.2)	21 (26.2)
Hiring non-clinician staff only	23 (60.5)	20 (47.6)	43 (53.8)
Not hiring	4 (10.5)	10 (23.8)	14 (17.5)
Unsure	1 (2.7)	1 (2.4)	2 (2.5)
Survey respondent role			
Physician/Advanced Practice Provider	22 (57.9)	31 (73.8)	53 (66.3)
Behavioral Health/Social Work	0 (0)	5 (11.9)	4 (5.0)
Nurse/Medical Assistant	7 (18.4)	4 (9.5)	11 (13.8)
Clinic Manager	5 (13.2)	2 (4.8)	7 (8.7)
Other	4 (10.5)	0 (0)	5 (6.2)

*Clinician included any of the following: Behavioral health provider, Community health worker (CHW), Front office staff, Medical assistant, Medical director or chief medical officer, Nurse (RN, LPN), Nurse practitioner or physician assistant, Office or clinic manager, Physician- Family Medicine, Physician- Internal Medicine, Physician- Pediatrics, Physician- Women’s health, Physician-other, Quality improvement specialist, Other

Table 2 summarizes impacts of the vaccine mandate on staffing, comparing rural and urban settings. About half of clinics reported losing staff: 53% of rural clinics and 41% of urban clinics. There was a statistically significant difference in the use of vaccination waivers (religious or medical) at rural clinics compared to urban clinics (71% versus 33.3%, p<0.004). Many clinics (60%) reported that staff became newly vaccinated after the statewide mandate. More rural clinics reported significant (34%) or very significant (11%) impacts compared to urban clinics (significant = 19% or very significant = 2%; p = 0.04). Ratios demonstrated this same pattern with trends toward more job loss, more waivers, and more new vaccinations in rural clinics, though only the difference in vaccination waivers achieved statistical significance.

**Table 2:** Impacts of COVID-19 vaccine mandate on rural versus urban clinics.

Outcomes	Rural Clinics N = 38	Urban Clinics N = 42	Total Clinics N = 80	
*Categorical & Ordinal Data*	*n (%)*	*n (%)*	*n (%)*	p-value*
Job loss				*0*.*547*
Jobs lost	20 (52.6)	17 (40.5)	37 (46.3)	
No jobs lost	16 (42.1)	21 (50.0)	37 (46.3)	
Unsure	2 (5.3)	4 (9.5)	6 (7.6)	
Waivers				***<0*.*004***
Staff or clinicians received waivers	27 (71.1)	14 (33.3)	41 (51.3)	
No staff or clinicians received waivers	8 (21.0)	19 (45.3)	27 (33.7)	
Unsure	3 (7.9)	9 (21.4)	12 (15.0)	
Newly vaccinated				*0*.*102*
Staff newly vaccinated	27 (71.1)	21 (50.0)	48 (60.0)	
No staff newly vaccinated	8 (21.0)	11 (26.2)	19 (23.8)	
Unsure	3 (7.9)	10 (23.8)	13 (16.2)	
Perceived overall impact on clinic staffing				***0*.*048***
No impact	7 (18.4)	21 (50.0)	28 (35)	
Minor impact	14 (36.8)	12 (28.6)	26 (32.5)	
Significant impact	13 (34.2)	8 (19.0)	21 (26.3)	
Very significant impact	4 (10.5)	1 (2.4)	5 (6.3)	
*Ratios*	*Mean (SD)*	*Mean (SD)*	*Mean (SD)*	*p-value* ±
Jobs lost per number of clinicians	0.38 (0.76)	0.17 (0.26)	0.27 (0.57)	*0*.*123*
Vaccine waivers per number of clinicians	0.99 (1.04)	0.28 (1.00)	0.60 (1.07)	***0*.*013***
Staff newly vaccinated per number of clinicians	0.64 (1.68)	0.31 (0.54)	0.45 (1.17)	*0*.*391*

* Job losses, waivers, and vaccination compared using Freeman-Holman Extension of Fisher’s Exact Test. Perceived clinic impact compared using Mann-Whitney U Test.

± Two-sample t-test

^#^ Do not add to 100.0% due to rounding

Among 37 clinics reporting job loss as a result of the statewide vaccination mandate, almost 60% reported only 1 or 2 jobs were lost, but 2 of these 37 clinics reported >10 jobs lost: 31% lost front office positions, 27% lost medical assistants, 13% lost nurses, and 9% lost physicians or APPs.

### Qualitative results

Three major themes emerged in response to open-ended questions regarding clinic impacts of the vaccination mandate: (1) decline in clinic morale, (2) small but impactful effects on provision of patient care, and (3) mixed opinions of the vaccination mandate despite broad support for COVID-19 vaccination.

#### Decline in clinic morale

While a few clinics saw staff morale improve after the mandate, the majority of respondents noted increased stress and burnout after the mandate. This was reported to add to already high stress levels related to general COVID-19 care provision impacts. Staffing impacts associated with the mandate were most often noted as the greatest source of stress or morale decline. System-level policy decisions around waiver acceptance were also an important source of stress, as policies varied widely and were regarded as opaque to staff.

*“It has required those of us who remain to work harder*, *more hours*, *and try to cover for those who were terminated*. *Feelings of overwork*, *stress and burnout are very high*.*” -Rural Physician*

#### Impacts to provision of patient care

Staffing pressures strained clinics’ ability to provide high-quality patient care. “Everyone has been asked to do more with less,” according to one respondent. For some clinics, the mandate exacerbated pre-existing staffing shortages to the extent that they were “no longer able to support well visit, injections, [and] paps [i.e., Papanicolaou test],” and had “limited [blood pressure] checks, COVID testing.” At other clinics, physicians and APPs “had to do triage work in addition to seeing patients,” causing “significantly decreased provider appointments.” Several respondents described increased use of virtual appointments due to limited support staff in the clinics. A small number of respondents described a decline in the quality of patient care.

*“The loss of our functional staff and the change in our work dynamic has affected the care that we provide our patients in a way that has negatively impacted not only our staff*, *hospital*, *patients*, *and morale*, *but the community as a whole*. *Living in a rural area*, *this has had a rippling effect that will not be easily recovered from*.*” -Rural Clinical Staff*

Although most respondents did not report a negative impact in healthcare, they did note that this “required flexibility among leadership and remaining staff to ensure necessary coverage [was] available.”

#### Mixed opinions of the vaccination mandate

Respondents overwhelmingly noted support for vaccination against COVID-19, but had mixed thoughts about the mandate. Responses varied by staff role in the clinics–most physicians and APPs made positive comments about the vaccination mandate, while only about half of non-clinician staff commented positively. Regardless of their overall impressions of the mandate, a majority of respondents expressed frustration about its implementation at the local, health system, or state levels. Comments included concerns about communication (“This shows how tricky science communication can be”), exemptions (“There has been little guidance regarding what level of accommodation should be provided for those with approved exemptions”) and enforcement (“I am very discouraged by employers who choose not to enforce it”).

## Discussion

Our study found that 60% of clinics reported that staff became newly vaccinated after the statewide mandate, suggesting that the policy was successful in the primary care setting. Previous research supports the success of COVID-19 vaccine mandates on increased vaccination in other settings [25, 42, 43]. That said, about half of clinics reported losing staff, which is problematic for an already strained healthcare system, especially for small clinics and those in rural areas.

Respondents from rural clinics reported more job loss and more negative perceived staffing impacts–findings which may relate to clinic size/structure or pre-existing staffing challenges [44]. These negative impacts may be especially pronounced when sustained acutely during a time of unprecedented pre-existing healthcare strain. We also found significantly higher use of vaccination waivers (religious or medical) at rural compared to urban clinics, which suggests a potential systematic disparity in policy implementation and differences in pre-mandate regional vaccination rates, which have been reported in rural areas broadly [45]. As the loss of staff was not significantly different between rural and urban clinics, but the use of vaccination waivers was higher in rural clinics, waiver use may have allowed rural clinics to lose fewer staff than they likely would have without the option. With substantial and compounding impacts on small and often rural primary care clinics, future vaccine policies for HCP should consider taking into account setting (i.e., rural vs. urban), increasing the timeline to allow clinics to prepare for implementation, and carefully considering waiver use.

Respondents said that the vaccination mandate contributed to a decline in clinic morale, had small but impactful effects on patient care, and created tension between support of vaccination and challenges with implementation of the vaccination mandate. A previous study also reported tension between supporting vaccines in general and implementation of the COVID-19 vaccine mandate specifically [46]. This tension may be seen with novel viruses and future vaccinations, as people could view new vaccine products differently than familiar ones. Strategies that provide time to discuss vaccines, address individual concerns, and respect diverse beliefs may lead to increased acceptance and decreased impact on morale and tensions around implementation [47]. These specific strategies may also help assuage some common reasons for vaccination hesitancy among HCP including concerns about safety and efficacy [3], trust [48], and personal right infringement [49].

We applied observed staffing outcome ratios to a hypothetical medium-sized clinic with recommended primary care medical home staffing ratios [50] to consider the clinic-level impacts of the vaccination mandate. This hypothetical medium-sized clinic would have 5 physicians, 2 APPs, 7 medical assistants, and 7 administrative staff. Based upon the average overall observed job loss per clinician ratio of 0.27, this medium-sized clinic would have lost approximately 2 staff (9.5% of staff). Based on the observed proportions of job types lost, this clinic likely lost a medical assistant and a patient access specialist. A similar hypothetical medium-sized *rural* Oregon clinic with the same makeup (based on an average job loss per clinician ratio of 0.38) would have lost close to 3 jobs (14.3% of staff) after the vaccination mandate, perhaps 2 medical assistants and a clinic scheduler.

Based on an overall average waiver per clinician ratio of 0.60, the same hypothetical medium-sized Oregon primary care clinic would have granted 4 vaccination waivers (19% of staff). A hypothetical *rural* Oregon clinic with the same make-up (based on an average rural waiver per clinician ratio of 0.99) would have granted approximately 7 waivers (33.3% of staff). This example highlights a much higher proportion of job loss in this study (nearly 10%) compared to previous literature noting 0–3% after influenza vaccine mandates [7, 24]. This difference in job loss may reflect differential acceptability of the COVID-19 vaccine compared to influenza, differences in hospital-based compared to ambulatory populations, or regional differences unique to Oregon. This case example also demonstrates the high prevalence of vaccination waivers, particularly among rural clinics. Because these examples are extrapolated from averages (overall and among rural clinics), they do not represent the experiences of all Oregon primary care clinics, which were more variable. In the future, employment data and/or vaccine registry data may provide a more accurate view of vaccination in this population.

### Limitations

This cross-sectional survey study of Oregon primary care clinics and staff in our statewide practice-based research network rapidly reached clinics from a geographically diverse area. Response rate was approximately 30% for ORPRN member clinics (80/296) and 11% for all primary care clinics in Oregon (80/732). By relying on a limited sample from a single time-point in a single state, it may suffer from selection bias, lack of generalizability outside of Oregon, and findings limited to the survey timepoint immediately after policy implementation. As a survey, it may also suffer from recall bias and misclassification, though this is expected to be non-differential between urban and rural clinics and may simply dilute findings. With widely varied experiences from a modest number of clinics, the sample may have been underpowered to detect statistically and clinically significant differences based on rurality; and as an unadjusted analysis, the association between outcomes and rurality should be interpreted cautiously as they may reflect an element of confounding, particularly by characteristics unmeasured by our brief survey. Due to missing outcome data, 14% of responding clinics were excluded. A comparison of excluded versus included clinics showed that most incomplete surveys came from rural clinics (11 of 13) and more respondents of incomplete surveys (compared to complete) were clinic managers and not clinicians (see data table in S2 Appendix). This may reflect differences in the acceptability of this survey among rural clinics and/or administrative staff. Despite these limitations, study results provide important insights about vaccine policy mandates on rural and urban primary care clinics that can be used for future vaccine policy decision-making.

## Conclusions

With the continued pandemic and an anticipated increase in novel disease outbreaks and emerging vaccines, vaccination policies for HCP should consider potential negative staffing impacts and strategies to mitigate these effects to preserve healthcare access and avoid compounding rural disparities in care.

## Supporting information

S1 Appendix COVID-19 vaccine mandate primary care impacts survey. (DOCX)

S2 Appendix Table comparing incomplete vs. complete surveys. (DOCX)
