# Supplementary material for: Impact of the COVID-19 vaccination mandate on the primary care workforce and differences between rural and urban settings to inform future policy decision-making
Source: PLoS One. 2023 Jun 27;18(6):e0287553. doi: 10.1371/journal.pone.0287553 (PMC10298794; doi:10.1371/journal.pone.0287553)
Supplement: S2 Appendix — (DOCX) [file pone.0287553.s002.docx]

**Appendix 2. Table Comparing Incomplete* vs. Complete Surveys**

|  | Incomplete Surveys  N=13 (%) | Complete Surveys  N=80 (%) |
| --- | --- | --- |
| Location |  |  |
| Rural | 11 (84.6) | 38 (47.5) |
| Urban | 2 (15.4) | 42 (53.5) |
| Clinic size |  |  |
| Small (0-5 providers) | 4 (30.8) | 27 (33.8) |
| Medium (6-10 providers) | 2 (15.4) | 23 (28.8) |
| Large (11+ providers) | 7 (53.8) | 30 (37.5) |
| Survey Respondent Role |  |  |
| Physician/Advanced Practice Provider | 6 (46.2) | 53 (66.3) |
| Behavioral Health/Social Work | 2 (15.4) | 4 (5.0) |
| Nurse/Medical Assistant | 0 (0) | 11 (13.8) |
| Office/Clinic Manager | 5 (38.4) | 7 (8.7) |
| Other | 0 (0) | 5 (6.2) |

*Incomplete surveys were defined as missing a response for two or more primary outcomes (jobs lost, vaccination waivers granted, staff newly vaccinated, clinic impact)

**Appendix 2. COVID-19 Vaccine Mandate Primary Care Impacts Survey**

The COVID-19 pandemic has affected primary care practices in many different ways. One recent impact has been the COVID-19 vaccination statewide mandate for health care workers (Oregon Administrative Rule (OAR) 333-019-1010, COVID-19 Vaccination Requirement for Healthcare Providers and Healthcare Staff in Healthcare Settings).

We are interested in how this rule has affected staffing in your primary care clinic or health center. Please answer the following questions about staffing at your clinic. All answers will be confidential. In order to assure that clinic

responses are not duplicated, we are asking each location to indicate their clinic name.

Name of clinic or health center

__________________________________

Zip code of the clinic

__________________________________

How many primary care clinicians (MD, DO, NP, or PA) does your practice have?

__________________________________

Your role (check all that apply):

Behavioral health provider

Community health worker (CHW)

Front office staff

Medical assistant

Medical director or chief medical officer

Nurse (RN, LPN)

Nurse practitioner or physician assistant

Office or clinic manager

Physician- Family Medicine,

Physician- Internal Medicine

Physician- Pediatrics

Physician- Women's health

Physician-other

Quality improvement specialist

Other

Please specify "Physician-other"

__________________________________

Please specify "other"

__________________________________

Have staff or providers at your clinic resigned, been put on leave, or been terminated because they did not receive

the COVID-19 vaccine or an approved waiver of exemption?

Yes

No

Don't know

How many individuals in your clinic have resigned, put on leave, or been terminated related to the COVID-19 vaccine mandate? ______

What are the positions of those individuals who have resigned, been put on leave, or been

terminated?

Behavioral health provider

Community health worker (CHW)

Front office staff

Medical assistant

Medical director or chief medical officer

Nurse (RN, LPN)

Nurse practitioner or physician assistant

Office or clinic manager

Physician- Family Medicine

Physician- Internal Medicine

Physician- Pediatrics

Physician- Women's health

Physician-other

Quality improvement specialist

Other

Don't know

Do any of your staff or providers have an approved waiver of exemption for remaining unvaccinated for COVID-19

(SARS-CoV-2)?

Yes

No

Don't know

How many of your staff or providers have an approved waiver? ______

How many of the waivers are for medical reasons? ______

Was your clinic hiring for any positions in August or September, 2021?

Yes

No

Don't know

Identify which positions were open in August or September 2021 (please check all that apply):

Behavioral health provider

Community health worker (CHW)

Front office staff

Medical assistant

Medical director or chief medical officer

Nurse (RN, LPN)

Nurse practitioner or physician assistant

Office or clinic manager

Physician-Family Medicine

Physician-Internal Medicine

Physician-Pediatrics

Physician-Women's health

Physician-other

Quality improvement specialist

Other

Don't know

To your knowledge, did any staff at your clinic receive the COVID-19 vaccine between August 19th (the

announcement of the COVID-19 vaccine mandate) and October 18th (the date the mandate went into effect)?

Yes, some staff did get vaccinated during this time

No, to my knowledge no staff members got vaccinated during this time

I don't know whether any staff members got vaccinated during this time

How many staff got vaccinated for COVID-19 between August 19th and October 18th? ______

Overall, how significant of an impact has the COVID-19 vaccine mandate had on staffing at your clinic?

No impact

Minor impact

Significant impact

Very significant impact

How would you describe the effect of the COVID-19 healthcare vaccine mandate on the work experience for staff and providers at your clinic?

How would you describe the effect of the COVID-19 healthcare vaccine mandate on your clinic's ability to provide clinical care (e.g., type of services available, appointment availability, etc.)?

What other thoughts would you like to share about the COVID-19 vaccination mandate for healthcare workers in your clinic and community?

If you would be willing to be contacted to provide clarifying information, please include your name and email

address:

Yes

No

Name:

__________________________________

Email

__________________________________
